# Dapagliflozin Attenuates Hyperglycemia Related Osteoporosis in ZDF Rats by Alleviating Hypercalciuria

**DOI:** 10.3389/fendo.2019.00700

**Published:** 2019-11-05

**Authors:** Ji-Yu Wang, Yan-Zhen Cheng, Shuang-Li Yang, Min An, Hua Zhang, Hong Chen, Li Yang

**Affiliations:** ^1^Department of Endocrinology and Metabolism, Zhujiang Hospital, Southern Medical University, Guangzhou, China; ^2^Department of Endocrinology and Metabolism, The Second Affiliated Hospital of GuiZhou Medical University, Kaili, China

**Keywords:** dapagliflozin, bone microarchitecture, ZDF rats, type 2 diabetes mellitus, Sodium dependent glucose transporters 2 inhibitor

## Abstract

Recent studies showed that in patients with type 2 diabetes mellitus (T2DM), Sodium-dependent glucose transporters 2 inhibitor (SGLT2I) may cause potential adverse effects on skeleton such as increasing the risk of fracture. This risk is possibly mediated by effects induced by all SGLT2I class drugs but whether Dapagliflozin aggravates osteoporosis in patients with T2DM remains controversial. Therefore, we designed this study to explore how Dapagliflozin affects the metabolism and the quality of bone in. T2DM animal models The effect of Dapagliflozin on skeleton was evaluated on male ZDF (Zucker Diabetic Fatty) rats—a rat model of diet induced spontaneous T2DM. Dapagliflozin was administrated *via* gavage at the dosage of 1.0 mg/kg/day. Bone tissue mineral density and the microarchitecture of tibiae were measured with micro-CT and biomechanics characteristic of the femora were tested using a three-point bending test. Serum bone biomarkers and other metabolic parameters were also tested *via* ELISA or other assays. Our results found that diabetic rats demonstrated symptoms of osteoporosis and Dapagliflozin could help to alleviate these defections caused by diabetes. Compared to the negative controls, the serum CT (calcitonin) level in ZDF rats as well as the uric calcium and phosphate levels were elevated, and these symptoms were alleviated by Dapagliflozin. Tibiae of Dapagliflozin treated rats demonstrated decreased cortical tissue mineral density while trabecular tissue mineral density and mean bone mineral density received a rise when compared to the matched controls. ZDF rats also showed defections in femora stiffness which could be relieved by Dapagliflozin administration. The mechanism of Dapagliflozin affecting bone quality is possibly connected to the suppression of serum calcitonin and excretion of calcium *via* urine rose by hyperglycemia. In conclusion, Dapagliflozin can prevent osteoporosis in ZDF rats by alleviating hypercalciuria.

## Introduction

Diabetes mellitus describes a group of metabolic disorders characterized by increased blood glucose concentration. The global prevalence of diabetes in adults has increased dramatically over recent decades. According to the Diabetes Atlas published by the IDF (International Diabetes Federation), by 2040, global estimates of diabetes prevalence and health expenditure will reach 10.4% and 642 million USD, respectively (1). Long-term hyperglycemia and inadequate glycemic control both contribute to the development of diabetic complications, including diabetic related osteoporosis, characterized by a number of detrimental effects on bone metabolism, which has significant consequences for patients with diabetes in terms of decreased bone mineral density and increased risk of fractures (2). Diabetes related osteoporosis can increase the risk of fractures, which can impact the life quality and life span of diabetes patients adversely. Nowadays, the main treatment means of diabetes related osteoporosis are restricted to relieving hyperglycemia and providing enough calcium *via* food or drugs. Therefore, exploring new methods of treating diabetes related osteoporosis is crucial in the near future.

Until a few years ago, the possibility that glucose-lowering drugs can affect bone metabolism and the risk of fracture in patients with diabetes was not even considered. Although drugs for T2DM are prescribed for their effect on blood glucose, it has been discovered that some class of anti-hyperglycemic drugs possess abilities to affect bone metabolism either beneficially (as for Metformin or Irbesartan) or detrimentally (as for Thiazolidinediones) (3, 4). It is therefore necessary to study the risk and benefits brought on by anti-diabetic therapies. Dapagliflozin is an SGLT2I (sodium-dependent glucose co-transporter type 2 inhibitor) that was approved by the FDA (Food and Drug Administration) on 8 January 2014, to treat T2DM. By inhibiting the reabsorption of filtered glucose in the renal proximal tubule, this class of drugs lower blood glucose levels in patients with diabetes, independent of insulin action or secretion and with lower probability of inducing hypoglycemia (5). As a class of drug with a unique mechanism in controlling hyperglycemia, Dapagliflozin has attracted increased attention from researcher. In a study carried out on T2DM patients with moderate renal impairment, Dapagliflozin increased the risk of fractures (6). According to the document published by the FDA, SGLT2I treating can increase the serum levels of PTH and FGF23 and further damage the bone in diabetic patients (7). However, firm conclusions cannot be drawn because the patients in this study had already developed renal impairment and their hyperglycemia was uncontrolled. In the research carried out by Konstantinos, no difference in the risk of fragility fractures was detected between participants prescribed Dapagliflozin and the matched controls (8). Therefore, more studies are needed to determine the effects of Dapagliflozin on bone in patients with T2DM. To verify the effects of Dapagliflozin on bone quality and material strength under diabetic condition, we treated ZDF (Zucker Diabetic Fatty) rats, a spontaneous T2DM model, with Dapagliflozin for 9 weeks at a dosage of 1.0 mg/kg/day to investigate the impact on bone brought on by SGLT2I therapy in T2DM.

## Materials and Methods

### Animal Models

The ZDF rats and the ZLC (Zucker lean control) rats were purchased from the Laboratory Animal Center of Vital River [Beijing, China; license number, SYXK (Yue) 2011 0074]. The ZDF rats exhibit a propensity for the development of T2DM after the administration of a high-fat diet purina#5008 and ZLC rats were used as the non-diabetic controls (9). Seven ZLC rats and 14 Male ZDF rats, at 8 weeks of age, were treated with high-fat diet purina#5008 for 4 weeks to induce diabetes. At 12 weeks of age, diabetic rats, as confirmed by random blood glucose level ≥300 mg/dl (16.7 mmol/L), were then randomly assigned to the diabetic control group (ZDF, *n* = 6) or the treatment group (ZDF+DA, *n* = 8). High-fat diet purina#5008 was provided from 8 weeks of age to the end of the experiment. At the beginning of the 13th week, rats in the ZDF+DA group were medicated with Dapagliflozin (1.0 mg/kg, once a day) *via* gavage. Rats in the ZDF group, treated with a vehicle *via* gavage, were used as the diabetic control and rats in the ZLC group were used as the negative control. At 21 weeks of age, the rats were anesthetized and sacrificed.

### Body Weight, Serum Calcium, Phosphate, Glucose, and Lipid Metabolism

Blood glucose, and body weight were monitored at the same time once a week, and blood samples were taken from the tail vein for plasma glucose test by a glucose meter (ACCU-CHEK Active, Roche Diagnostics, Basel, Switzerland). An oral glucose tolerance test was conducted without anesthesia on the morning after an overnight fast at the end of week 20. After the Fasting blood glucose (FBG) test, rats were intragastrically given glucose solution at 2 g/kg body weight. The blood samples were collected from a tail vein for plasma glucose by a glucose meter at 0-, 30-, 60-, and 120-min following glucose administration. The AUC (area under curve) of glucose was calculated by the trapezoidal method (AUC = 1/4 fasting glucose + 1/2 30 min glucose + 3/4 60 min glucose + 1/2 120 min glucose). At 21 weeks of age, the rats were anesthetized by 2.5% pentobarbital sodium and the left ventricular was punctured for blood. The blood samples were used for the measurement of plasma glucose, HbA1c, total cholesterol (TC), triglycerides (TG), low-density lipoprotein cholesterol (LDL), high-density lipoprotein cholesterol (HDL), creatinine, calcium, and phosphate with an automatic biochemical analyzer (Aeroset, American). After blood was collected from the left ventricular, the left femur and tibia of both sides were harvested.

### Urine Volume, Urinary Calcium and Urinary Phosphate

To assess the consequences of SGLT2I-induced osmotic diuresis on urinary mineral loss, we also examined select components of calcium and phosphate homeostasis. Twenty-four-hour urine of each rat was collected, and the 24 h urine volume of each rat was measured 7 days before being sacrificed. Collected urine samples were stored at −20°C until further analysis. Urine collected from each animal 1 week before being sacrificed were used for urine calcium, urine phosphate and creatinine analyzation *via* biochemical analyzer (Aeroset, American). Urine calcium and phosphate concentration were then normalized to creatinine concentration and reported as a urine calcium/creatinine ratio (UCCR) or a urine phosphate/creatinine ratio (UPCR). All procedures were approved by the Institutional Animal Care and Use Committee at the Southern Medical University (Guangzhou, China).

### Micro-CT Assessment

The left tibia was immersed in 4% PFA (paraformaldehyde) at room temperature until being scanned. Bones were placed in a 24 mm specimen holder and scanned with a micro-computed tomography (micro-CT) scanner (Latheta LCT-200, Hitachi-Aloka Medical, Ltd., Tokyo, Japan). For all scans, the same parameters were used: The tube voltage was set at 50 kV, the current was constant at 1 mA and the resolution for scanning was 22 μm. A 2D scout view was used to select the region of interest (ROI): a 1.5 mm section of trabecular bone of the metaphysis and the cortical bone of diaphysis, beginning from 1 to 2.5 mm distal to the growth plate. The following parameters were analyzed using Analyze 12.0 (AnalyzeDirect, Inc. United States): Cortical tissue mineral density (Ct.TMD), Trabecular tissue mineral density (Tb.TMD), Mean bone mineral density (mean.BMD), Trabecular thickness (Tb.Th), Trabecular separation (Tb.Sp), Trabecular number (Tb.N), Cortical thickness (Ct.Th), Bone volume/tissue volume (BV/TV), Structural model index (SMI), Connectivity density (Conn.D). Standard evaluation scripts from the manufacturer were used to determine the architectural and structural properties of trabecular and cortical bone, respectively.

### Biomechanical Testing

The left femurs were frozen in 1× PBS (phosphate buffered saline) and stored at −20°C until analysis. Before biomechanical examination, the left femurs were slowly thawed for 8 h at room temperature while immersing in 1× PBS. To determine the mechanical properties of the cortical bone, each hydrated femur was loaded to failure at a speed of 2 mm/min using a three-point bending fixture (bending about the medial–lateral plane). The span between the lower support was 20 mm. From the resulting forces recorded by a material testing machine (Instron ElectroPuls, E1000, USA) and displacements recorded by the LVDT (Dynamight 8841, Instron; Canton, OH), structural properties included maximum displacement, fracture load, stiffness, and energy absorption were obtained based on the load–deformation curve.

### Bone Related Hormones and Biomarker Analyses

For all three groups, the following biomarkers and hormones in serum were measured at sacrifice *via* rat ELISA (enzyme linked immunosorbent assay) kits (Uscnlife, Wuhan EIAab Science Co., Ltd, Wuhan, China): procollagen type 1N-terminal propeptide (PINP), C-terminal telopeptides of type I collagen (CTX-I), rat bone alkaline phosphatase (BALP), Bone gla protein (BGP), calcitonin (CT), parathyrin (PTH). All these proteins were measured by ELISA kits according to the instructions of the manufacturer.

### Quantitative RT-PCR Assay

Total RNA was extracted from the right tibia using a Trizol reagent® (Invitrogen, Carlsbad, CA, USA) according to the manufacturer's instructions. To quantify disease or drug-induced changes in osteal expression of genes involved in calcium and phosphate regulation, quantitative RT-PCR was performed. Primers used are listed below (Table 1). RT-PCR conditions were denatured at 95°C for 5 min, followed by 28–35 cycles of 95°C for 30 s, annealed at 57°C for 30 s in a thermal cycle. Real-time PCR was performed with the Applied Biosystems® 7500 Real-Time PCR Systems (Life technology, Carlsbad, CA 92008 USA). The reaction mixtures were prepared using SYBR® Green PCR Master Mix (Life technology, Carlsbad, CA 92008 USA), 0.5 μM of each primer, 4 mM MgCl_2_ and 2 μl of cDNA in a final volume of 20 μl. The reaction condition consisted of denaturation at 95°C for 10 min, followed by 40 cycles of 95°C for 15 s and 64°C for 15 s, followed by a melting curve analysis. For each sample, PCR was performed in duplicate. The quantitative amount of each gene was normalized against the house-keeping gene glyceraldehyde-3-phosphate dehydrogenase (GAPDH).

**Table 1:** Primers used for RT-qPCR test.

**Gene**	**Sence**	**Anti-sence**
GAPDH	AGACAGCCGCATCTTCTTGT	TGATGGCAACAATGTCCACT
OSX	GGCTTTTCTGTGGCAAGAGGTT	CGCTGATGTTTGCTCAAGTGGTC
ALP	CCAGAAAGACACGTTGACTGTGG	TCTTGTCCGTGTCGCTCACCAT
OCN	AGCTCAACCCCAATTGTGAC	TCCTGGAGAGTAGCCAAAGC
OPN	GCTAAGCCTCAGCATCCTTG	AAGCAAACCACTGCCAGTCT

*GAPDH, glyceraldehyde-3-phosphate dehydrogenase; OCN, osteocalcin; OSX, Osterix; ALP, Alkaline Phosphatase; OPN, osteopontin*.

### Statistical Analyses

Statistical analysis was performed with the ANOVA using SPSS (version 19.0; SPSS Inc., Chicago, IL, USA). For each parameter, a step-down Tukey method was used to keep the overall family-wise error rate under 0.05, to adjust for multiple comparisons.

## Results

### Body Weight, Glucose and Lipid Metabolism

#### Effect of Dapagliflozin on Body Weight

**Figure 1:**
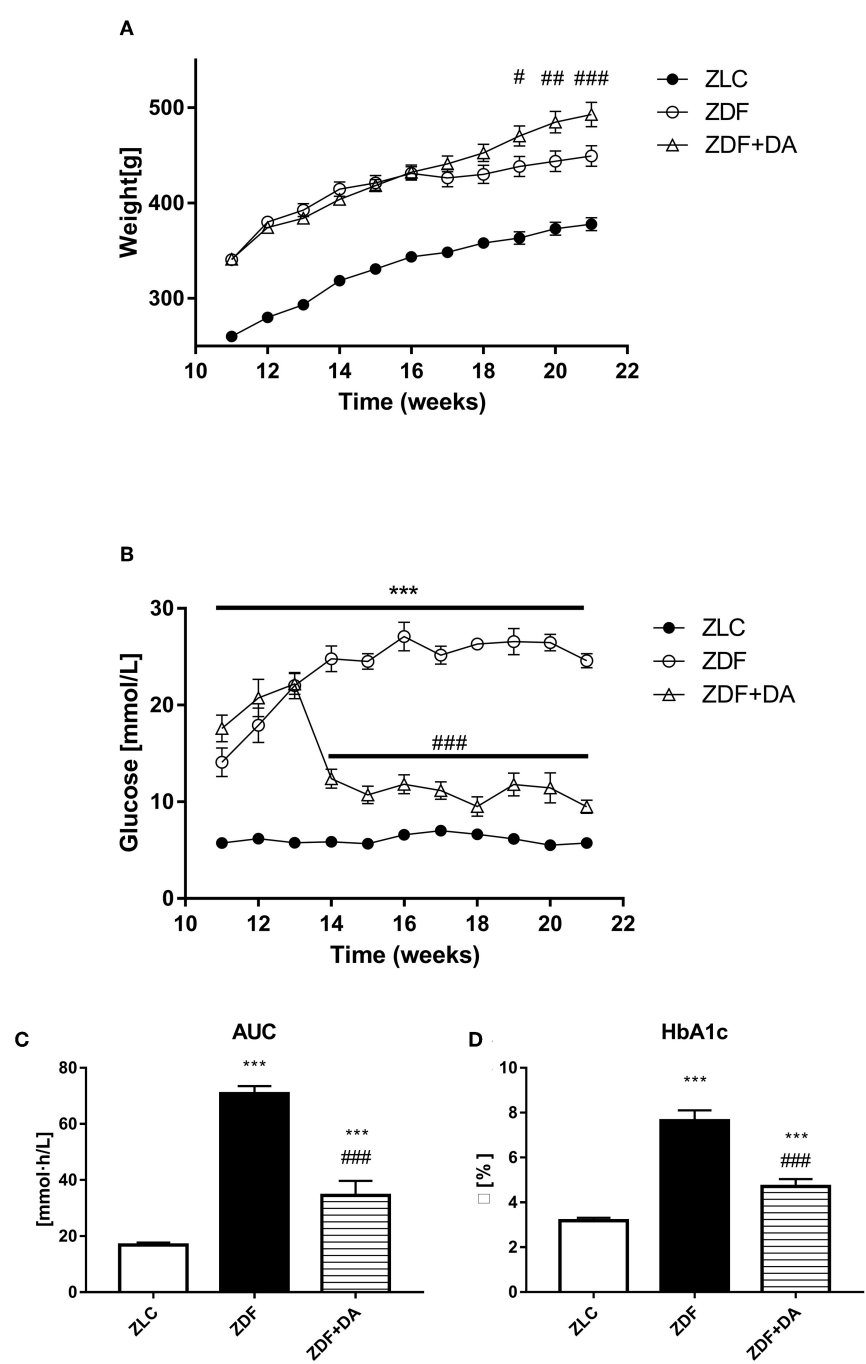
Body weight and glucose metabolism in rats with Dapagliflozin. Twelve-week-old rats were randomly assigned into two subgroups: ZDF rats treated with vehicle (ZDF); ZDF rats treated with Dapagliflozin (ZDF+DA, Dapagliflozin, 1 mg/kg, po. per day). **(A)** Body weight throughout the study period. **(B)** Blood glucose throughout the study period. **(C)** Blood glucose AUC at the end of 9-week treatment. **(D)** HbA1c at the end of 9-week treatment. Values are means ± SD. Statistically significant differences compared to ZLC group are indicated with asterisk (***p < 0.001). Statistically significant differences compared to ZDF group are indicated with octothorpe (^#^*p* < 0.05; ^##^*p* < 0.01; ^###^*p* < 0.001). AUC, area under curve; HbA1c, glycated hemoglobin.

As showed in Figure 1A, the mean body weight of the ZLC group (*n* = 7) is lower than both the ZDF group (*n* = 6, *P* < 0.001) and the ZDF+DA group (*n* = 8, *P* < 0.001) throughout the process of treatment. At the basal line, the mean body weight of the ZDF and ZDF+DA group showed no significant difference (ZDF vs. ZDF+DA, *p* > 0.05). The difference of body weight became significant at week 19 (*p* < 0.05). By the time of sacrifice, the mean body weight of the ZDF+DA group was the highest. (ZDF vs. ZDF+DA, *p* < 0.001).

#### Effect of Dapagliflozin on Glucose Metabolism

At the beginning of the 12th week, confirmed by a random glucose test, both groups of ZDF rats demonstrated symptoms of hyperglycemia (Figure 1B). After treatment with Dapagliflozin, as demonstrated in Figure 1B, the random glucose level was lower in the ZDF+DA group than in the ZDF group (ZDF, vs. ZDF+DA, *p* < 0.001). On the other hand, the glucose AUC of OGTT demonstrated impaired glucose tolerance in both the ZDF and ZDF+DA group (ZLC vs. ZDF, *p* < 0.001; ZLC vs. ZDF+DA, *p* < 0.001; ZDF vs. ZDF+DA, *p* < 0.001) (Figure 1C). At the end of the experiment, the HbA1c level in the ZDF group was higher than in the other two groups (ZLC vs. ZDF, *p* < 0.001; ZDF vs. ZDF+DA, *p* < 0.001). Dapagliflozin can lower HbA1c in diabetic rats but not enough to cure diabetes in ZDF rats (ZLC vs. ZDF+DA, *p* < 0.001) (Figure 1D).

#### Effect of Dapagliflozin on Lipid Metabolism

**Figure 2:**
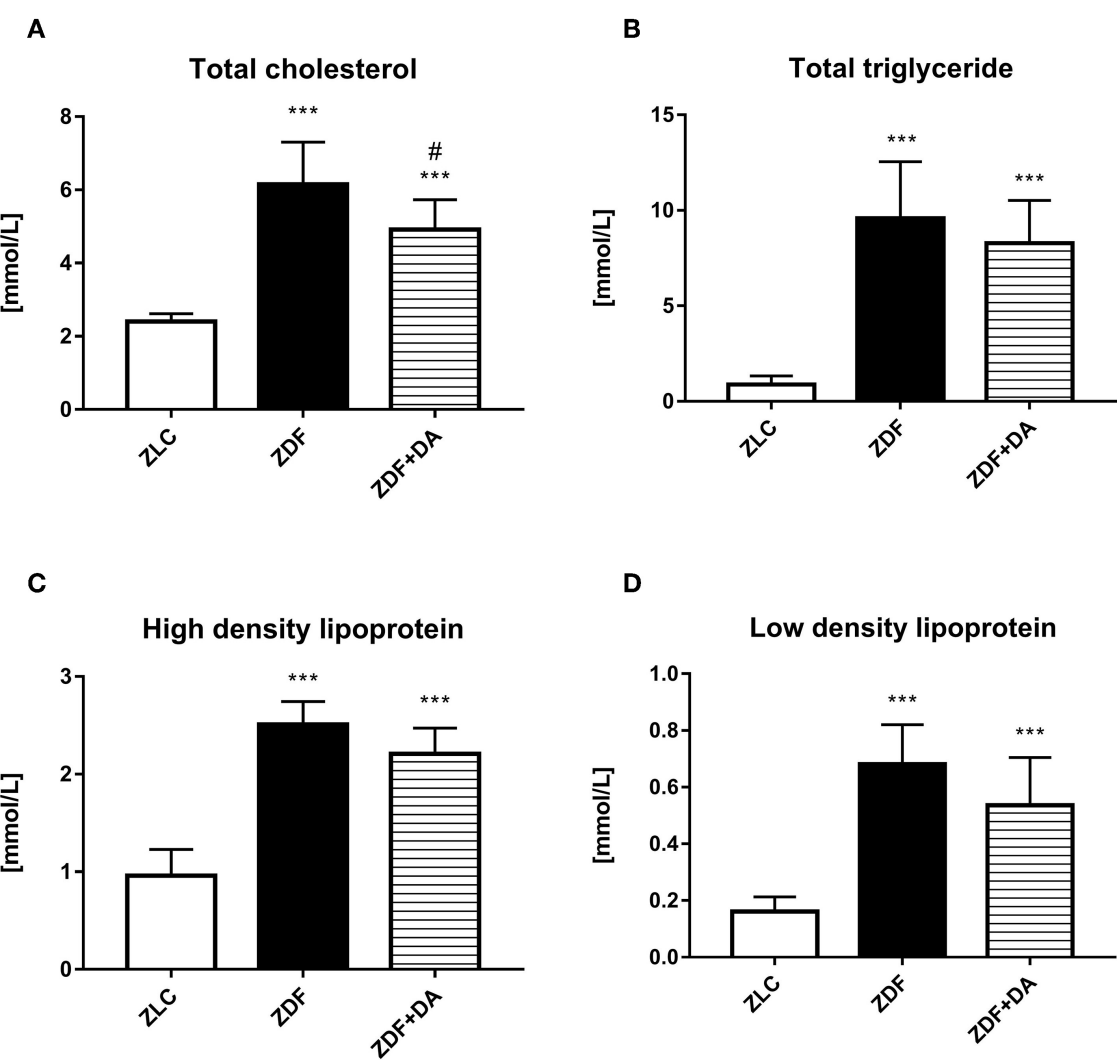
Lipid metabolism in rats with Dapagliflozin. 12-week-old rats were randomly assigned into two subgroups: ZDF rats treated with vehicle (ZDF); ZDF rats treated with Dapagliflozin (ZDF+DA, Dapagliflozin, 1 mg/kg, po. per day). **(A)** Serum total cholesterol level at the end of 9-week treatment. **(B)** Serum total triglyceride level at the end of 9-week treatment. **(C)** Serum high density lipoprotein level at the end of 9-week treatment. **(D)** Serum low density lipoprotein level at the end of 9-week treatment. Values are means ± SD. Statistically significant differences compared to ZLC group are indicated with asterisk (***p < 0.001). Statistically significant differences compared to ZDF group are indicated with octothorpe (^###^*p* < 0.001).

As for lipid metabolism, both ZDF and ZDF+DA groups demonstrated rise in levels of TC, TG, HDL, and LDL compared to the ZLC group (Figures 2A–D, ZLC vs. ZDF/ZDF+DA, *p* < 0.001). These changes indicated that diabetes rats demonstrated the symptom of lipid metabolism disturbance. However, even though the means of TC, TG, HDL, and LDL were lower in the ZDF+DA group compared to the ZDF group, it was only in TC that the difference between the ZDF and ZDF+DA group was significant (Figure 2A, ZDF+DA vs. ZDF, *p* < 0.05).

#### Effect of Dapagliflozin on Ion Metabolism, Urinary Volume and Creatinine

To evaluate the effect of Dapagliflozin on ion homeostasis, we measured the concentration of calcium and phosphate in both serum and urine. We also measured the urinary volume and creatinine to see if the kidney function of ZDF rats demonstrated any change after Dapagliflozin administration. Urine calcium and phosphate were adjusted according to the creatinine into the Urine calcium creatinine ratio (UCCR) and Urine phosphate creatinine ratio (UPCR).

**Figure 3:**
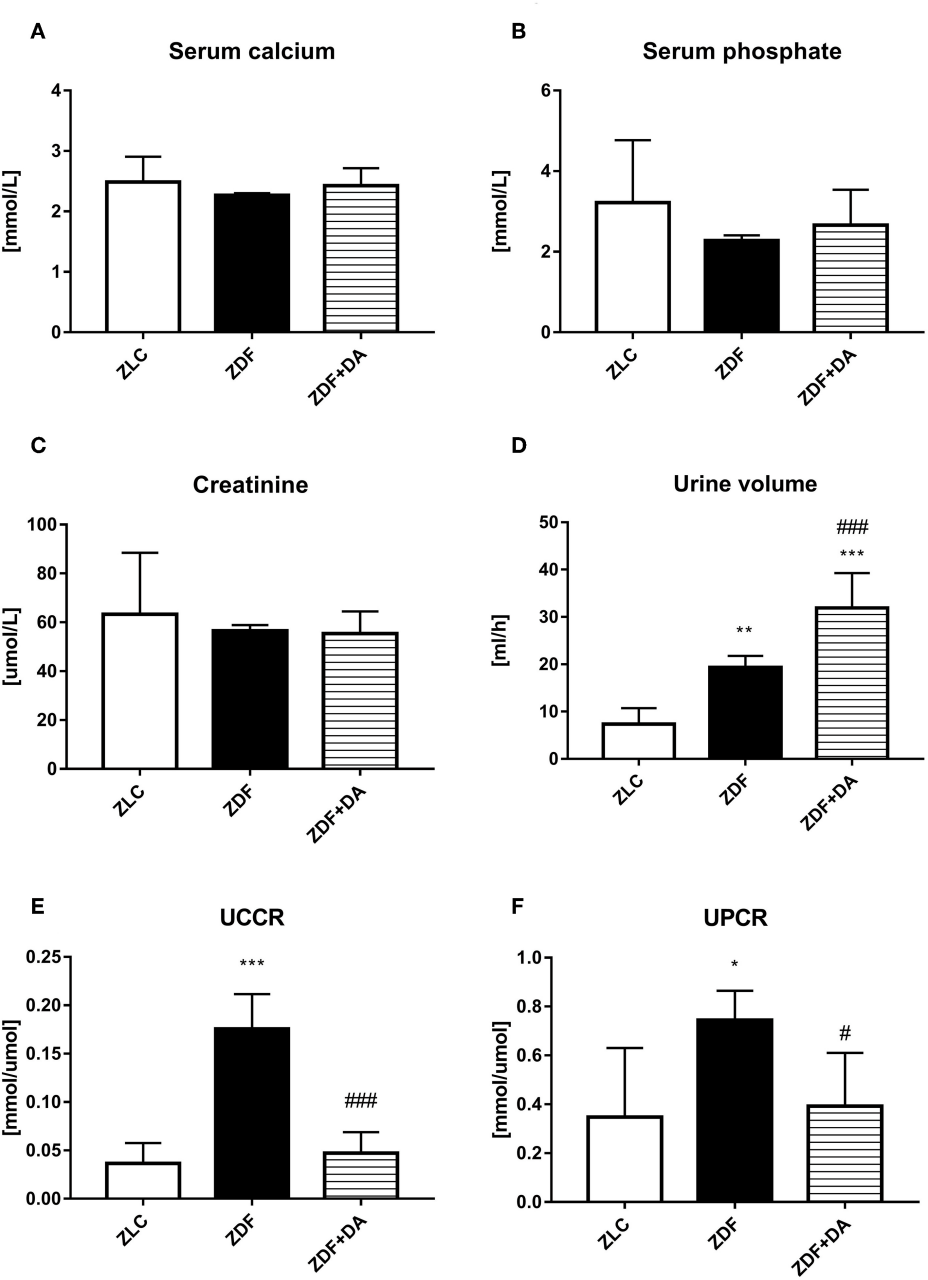
Creatinine, urine volume and ion metabolism in rats with Dapagliflozin. Twelve-week-old rats were randomly assigned into two subgroups: ZDF rats treated with vehicle (ZDF); ZDF rats treated with Dapagliflozin (ZDF+DA, Dapagliflozin, 1 mg/kg, po. per day). **(A)** Serum calcium level at the end of 9-week treatment. **(B)** Serum phosphate level at the end of 9-week treatment. **(C)** Serum Creatinine level at the end of 9-week treatment. **(D)** 24 h-urine volume one week before sacrifice. **(E)** UCCR at the end of 9-week treatment **(F)** UPCR at the end of 9-week treatment. Values are means ± SD. Statistically significant differences compared to ZLC group are indicated with asterisk (*p < 0.05; **p < 0.01; ***p < 0.001). Statistically significant differences compared to ZDF group are indicated with octothorpe (^#^*p* < 0.05; ^###^*p* < 0.001). Abbreviations: UCCR, urine calcium creatinine ratio; UPCR, urine phosphate creatinine ratio.

Diabetes and Dapagliflozin did not affect serum calcium level (Figure 3A, *p* > 0.05). The mean of serum phosphate and creatinine in the three groups demonstrated no significant difference (Figures 3B,C, *P* > 0.05). Diabetic rats (ZDF groups) tend to urinate more urine than normal rats (ZLC group). This symptom of hyperuresis was aggravated after the treatment of Dapagliflozin (Figure 3D, ZDF vs. ZLC, *P* < 0.01; ZDF+DA vs. ZDF, *P* < 0.001). Even though serum calcium and phosphate did not differ among the three groups, rats with diabetes (ZDF group) tend to excrete more calcium and phosphate *via* the kidney compared to non-diabetic rats (ZLC group). These symptoms of hypercalciuria and hyperphosphaturia can be alleviated by Dapagliflozin (Figures 3E,F, ZLC vs. ZDF, *p* < 0.05; ZLC vs. ZDF+DA, *p* < 0.05).

#### Micro-CT Scan

**Figure 4:**
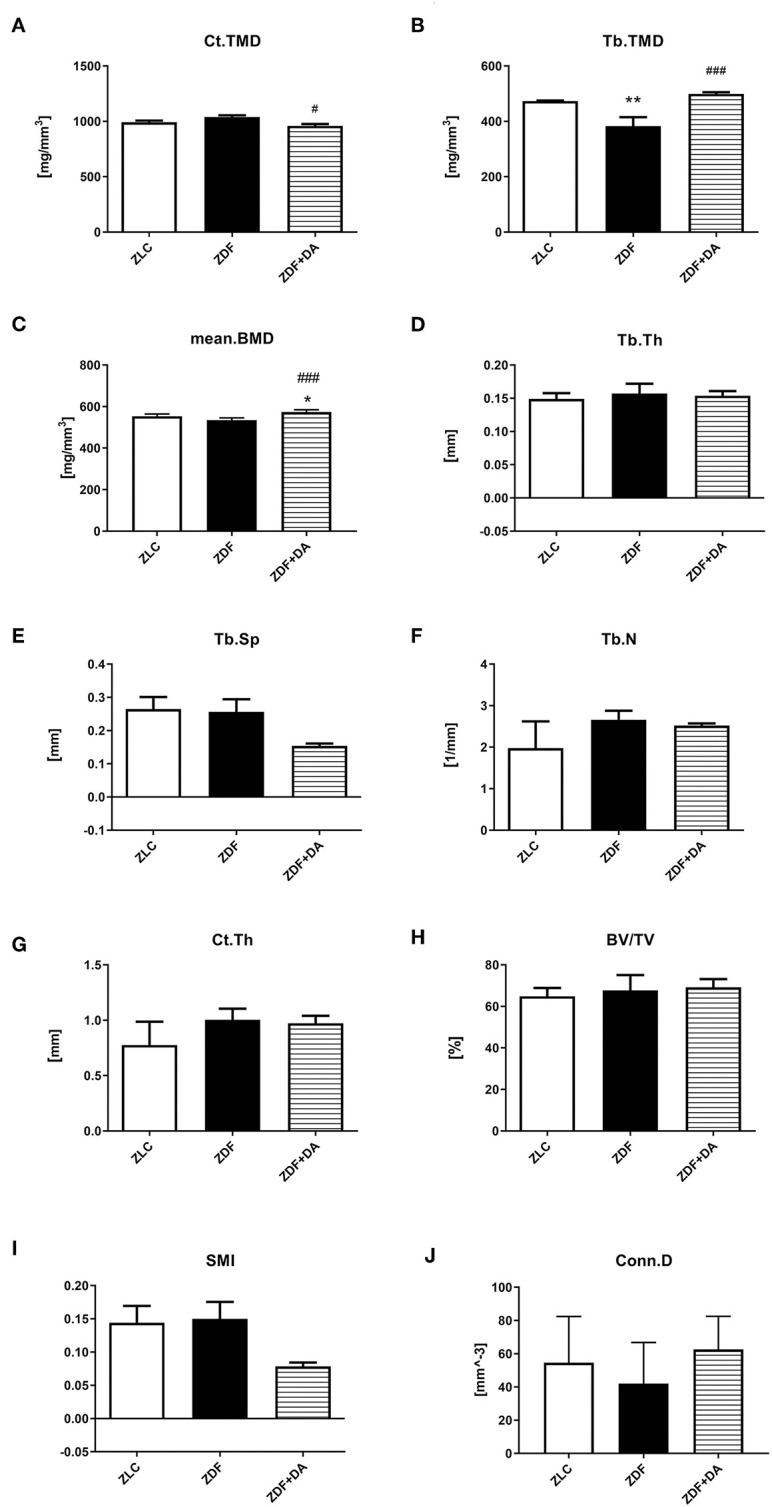
Micro-CT scan in rats with Dapagliflozin. Twelve-week-old rats were randomly assigned into two subgroups: ZDF rats treated with vehicle (ZDF); ZDF rats treated with Dapagliflozin (ZDF+DA, Dapagliflozin, 1 mg/kg, po. per day). **(A)** Ct.TMD at the end of 9-week treatment. **(B)** Tb.TMD at the end of 9-week treatment. **(C)** mean.BMD at the end of 9-week treatment. **(D)** Tb.Th at the end of 9-week treatment. **(E)** Tb.Sp at the end of 9-week treatment. **(F)** Tb.N at the end of 9-week treatment. **(G)** Ct.Th at the end of 9-week treatment. **(H)** BV/TV at the end of 9-week treatment. (I) SMI at the end of 9-week treatment. (J) Conn.D at the end of 9-week treatment. Values are means ± SD. Statistically significant differences compared to ZLC group are indicated with asterisk (*p < 0.05; **p < 0.01). Statistically significant differences compared to ZDF group are indicated with octothorpe (^#^*p* < 0.05; ^###^*p* < 0.001). Ct.TMD, cortical tissue mineral density; Tb.TMD, trabecular tissue mineral density; mean.BMD, mean bone mineral density; Tb.Th, trabecular thickness; Tb.Sp, trabecular separation; Tb.N, trabecular number; Ct.Th, cortical thickness; BV/TV, bone volume/tissue volume; SMI, structure model index; Conn.D, connectivity density.

**Figure 5:**
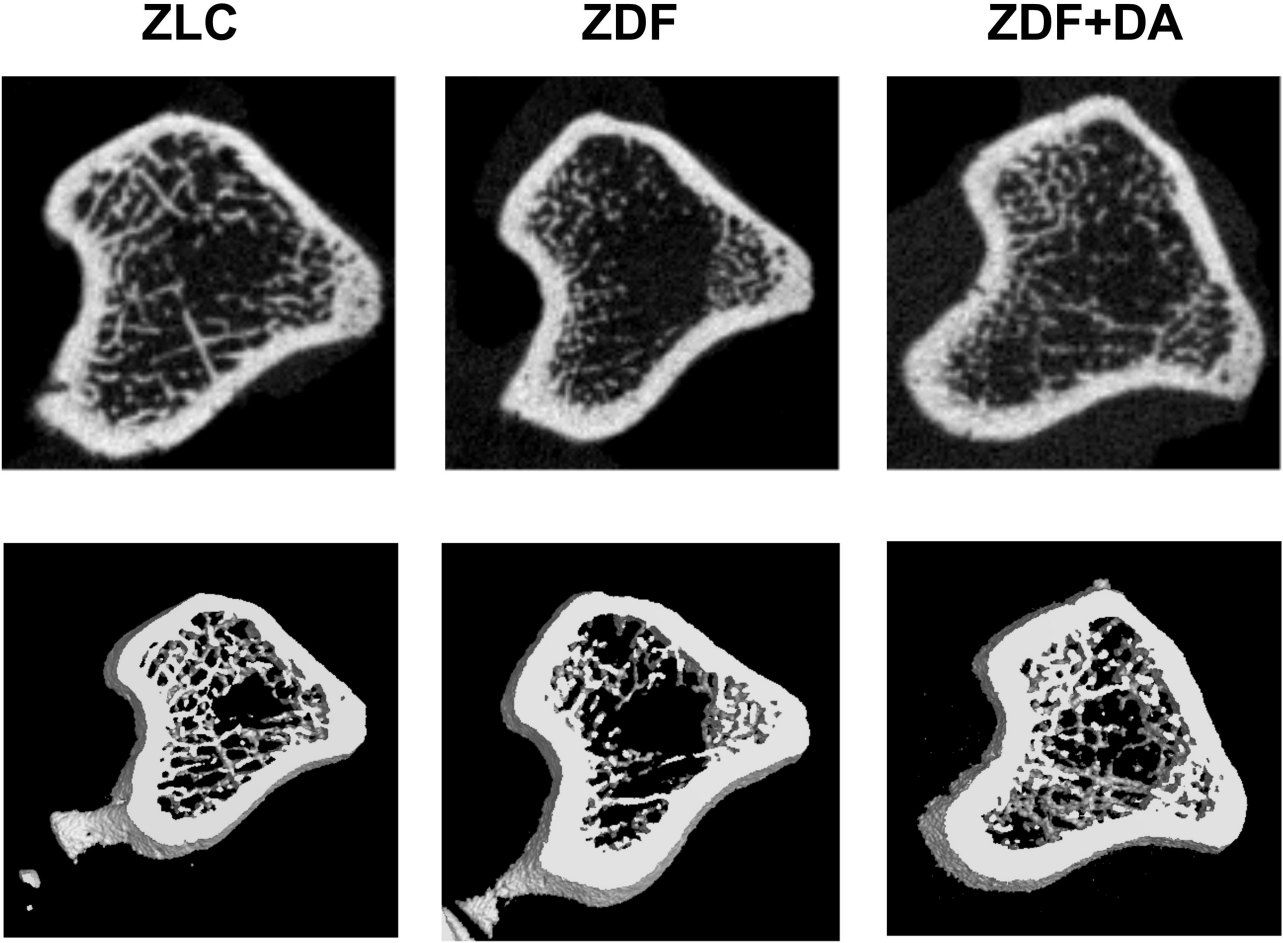
Representative micro-CT scan images. Twelve-week-old rats were randomly assigned into two subgroups: ZDF rats treated with vehicle (ZDF); ZDF rats treated with Dapagliflozin (ZDF+DA, Dapagliflozin, 1 mg/kg, po. per day). A representative micro-CT images from each of the three groups is shown.

In6 order to examine whether diabetes or Dapagliflozin affects the mineral density and the microarchitecture of the skeleton, we scanned the left tibiae of rats with a micro-CT. According to the results, diabetes can cause a decrease in Tb.TMD (Figure 4B, ZDF vs. ZLC, *p* < 0.01) but did not affect the density of cortical tissue (Figure 4A, ZDF vs. ZLC, *p* > 0.05) and the mean bone density (Figure 4C, ZDF vs. ZLC, *p* > 0.05). Dapagliflozin can reverse the defects in Tb.TMD caused by diabetes while decreasing Ct.TMD (Figures 4A,B, ZDF+DA vs. ZLC, *p* < 0.05; ZDF+DA vs. ZDF, *p* < 0.001), but mean BMD of tibia was increased after Dapagliflozin treatment (Figure 4C, ZDF+DA vs. ZDF, *p* < 0.001). However, neither diabetes nor Dapagliflozin affected bone microarchitecture parameters (Figures 4D–H, *p* > 0.05). Representative CT images of the three groups are shown in Figure 5A.

#### Biomechanical Testing

**Figure 6:**
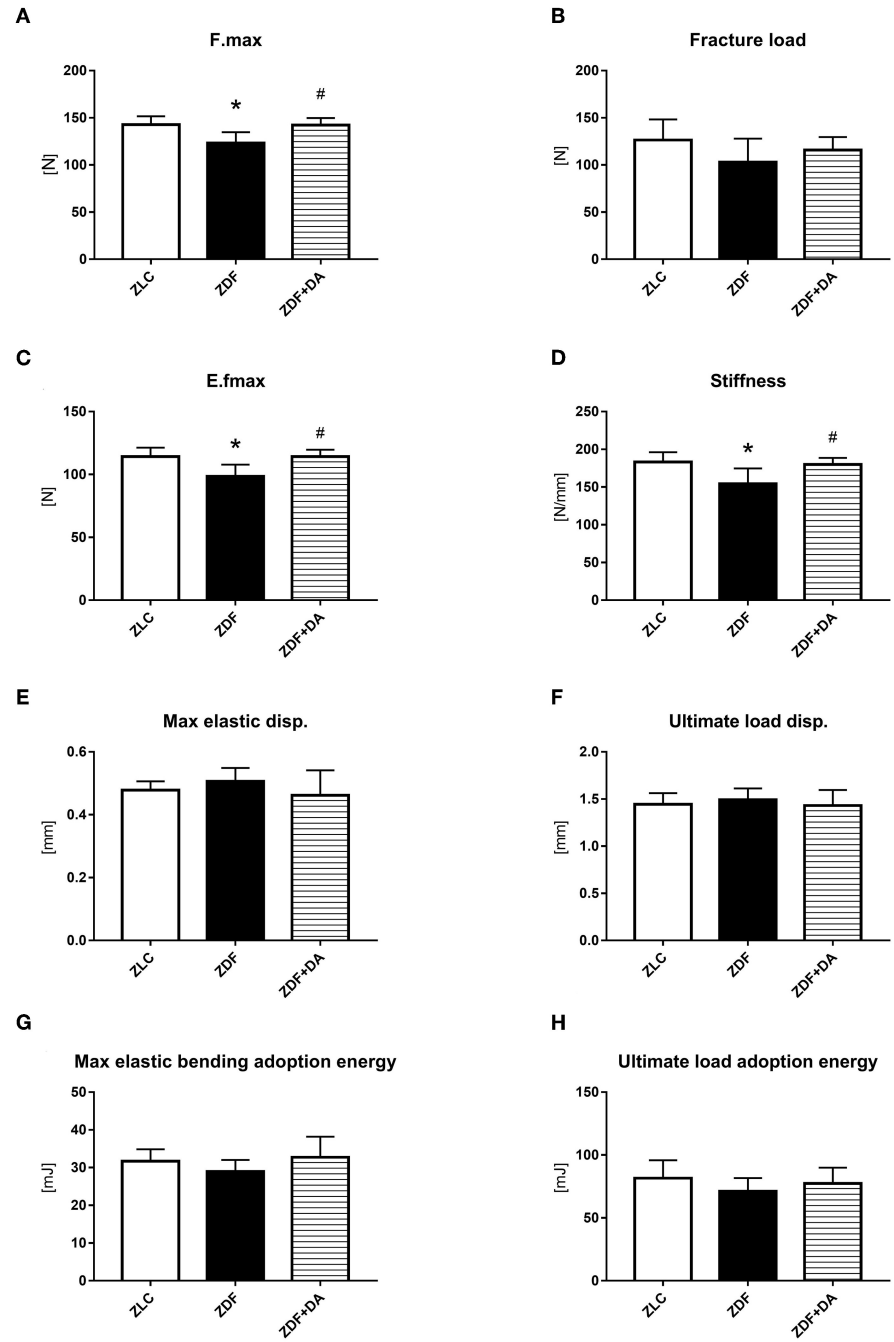
Biomechanical three-point bending testing. Twelve-week-old rats were randomly assigned into two subgroups: ZDF rats treated with vehicle (ZDF); ZDF rats treated with Dapagliflozin (ZDF+DA, Dapagliflozin, 1 mg/kg, po. per day). **(A)** F.max at the end of 9-week treatment. **(B)** Fracture load at the end of 9-week treatment. **(C)** E.fmax at the end of 9-week treatment. **(D)** Stiffness at the end of 9-week treatment. **(E)** Max elastic disp. at the end of 9-week treatment. **(F)** Ultimate load disp. at the end of 9-week treatment. **(G)** Max elastic bending adoption energy at the end of 9-week treatment. **(H)** Ultimate load adoption energy at the end of 9-week treatment. Values are means ± SD. Statistically significant differences compared to ZLC group are indicated with asterisk (*p < 0.05). Statistically significant differences compared to ZDF group are indicated with octothorpe (^#^*p* < 0.05). Abbreviations: F.max, maximal force; E.fmax, maximal elastic force; Max elastic disp., maximal elastic displacement; Ultimate load disp., ultimate load displacement.

Consistent with our finding in the micro-CT results, other researches also pointed out that long-term diabetes was associated with deficits in trabecular tissue mineral density (Tb.TMD, Figure 4B, *p* < 0.01) (9). To establish whether the defect of Tb.TMD in diabetes rats will affect bone strength, we performed a three-point bending test on the rat femurs. As a result, diabetes can damage the force resisting ability of the rat's femur (Figures 6A,C, ZDF vs. ZLC, *p* < 0.05), and lower the stiffness of the skeleton (Figure 6D, ZDF vs. ZLC, *p* < 0.05). However, diabetes did not affect the girder deflection (maximal elastic displacement, Figure 6E, ZDF vs. ZLC, *p* > 0.05) and energy adopted during the bending process (Figures 6G,H, ZDF vs. ZLC, *p* > 0.05). SGLT2I treatment can reverse the defections caused by diabetes (Figures 6A,C, ZDF+DA vs. ZDF, *p* < 0.05; ZDF+DA vs. ZLC, *p* > 0.05), and restore the stiffness of the skeleton (Figure 6D, ZDF+DA vs. ZDF, *p* < 0.05; ZDF+DA vs. ZLC, *p* > 0.05).

#### Bone Related Hormones and Biomarker Analyses via ELISA

**Figure 7:**
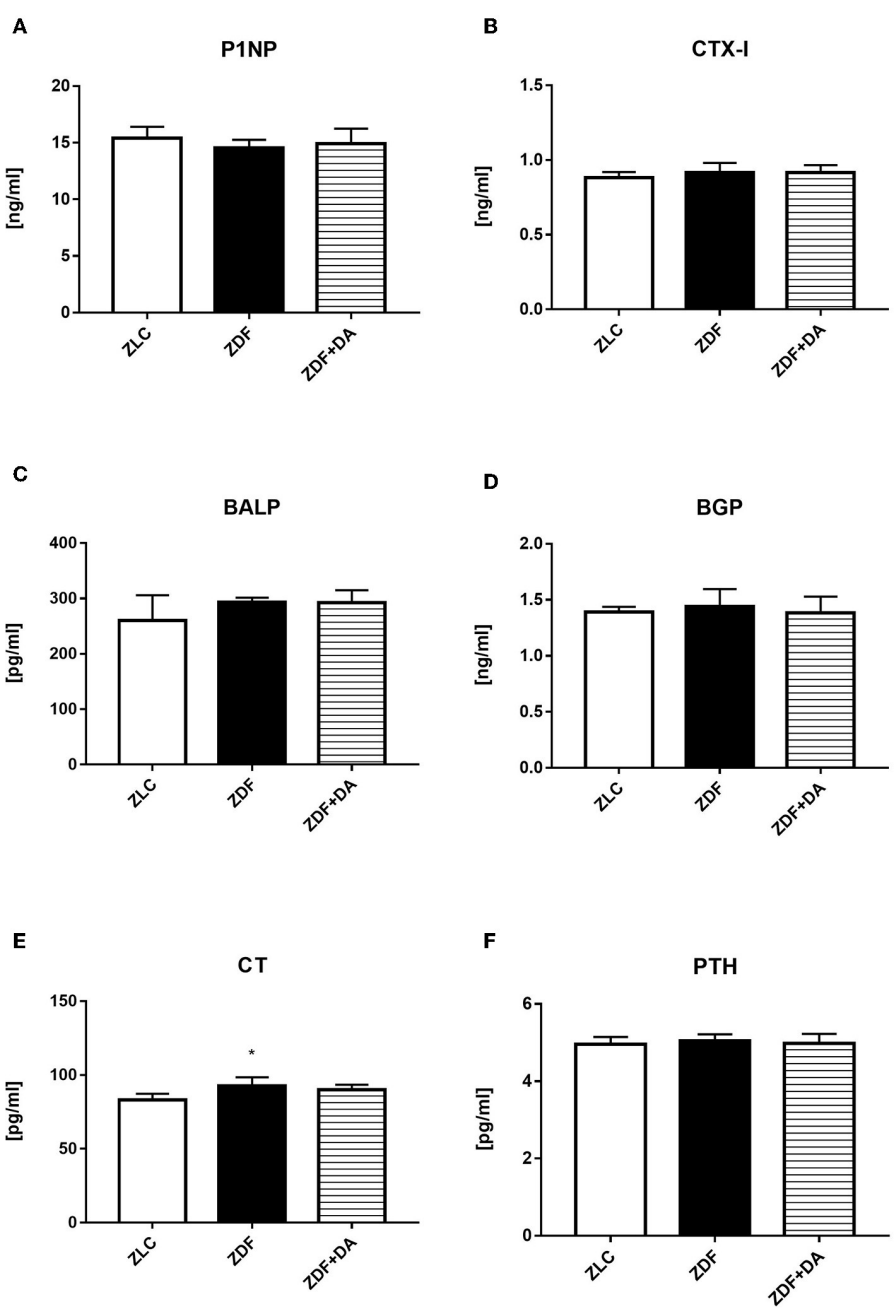
ELISA analyses on bone related hormones and biomarker. Twelve-week-old rats were randomly assigned into two subgroups: ZDF rats treated with vehicle (ZDF); ZDF rats treated with Dapagliflozin (ZDF+DA, Dapagliflozin, 1 mg/kg, po. per day). **(A)** PINP at the end of 9-week treatment. **(B)** CTX-I at the end of 9-week treatment. **(C)** BALP at the end of 9-week treatment. **(D)** BGP at the end of 9-week treatment. **(E)** CT at the end of 9-week treatment. **(F)** PTH at the end of 9-week treatment. Values are means ± SD. Statistically significant differences compared to ZLC group are indicated with asterisk (*p < 0.05). Abbreviations: PINP, procollagen type 1N-terminal propeptide; CTX-I, C-terminal telopeptides of type I collagen; BALP, bone alkaline phosphatase; BGP, Bone gla protein; CT, calcitonin; PTH, parathyrin.

In order to study how diabetes and SGLT2I affect bone *via* hormones and biomarkers, we tested PINP, a biomarker of bone formation; CTX-I, a biomarker of bone resorption; BALP, a hormone that indicates the level of bone calcification; BGP, a hormone that indicates the level of calcium deposition; and CT and PTH, hormones that affect calcium and phosphate metabolism. The CT level in the ZDF group was higher compared to the ZLC group and this elevation was suppressed by SGLT2I (Figure 7E, ZDF vs. ZLC, *p* < 0.05; ZDF+DA vs. ZLC, *p* > 0.05). This result is consistent with UCCR and UPCR results since CT can boost calcium and phosphate secretion *via* the kidney at the same time. The other hormones and biomarkers demonstrated no significant change among the three groups (Figures 7A–D,F, *p* > 0.05).

#### Quantitative RT-PCR Assay

**Figure 8:**
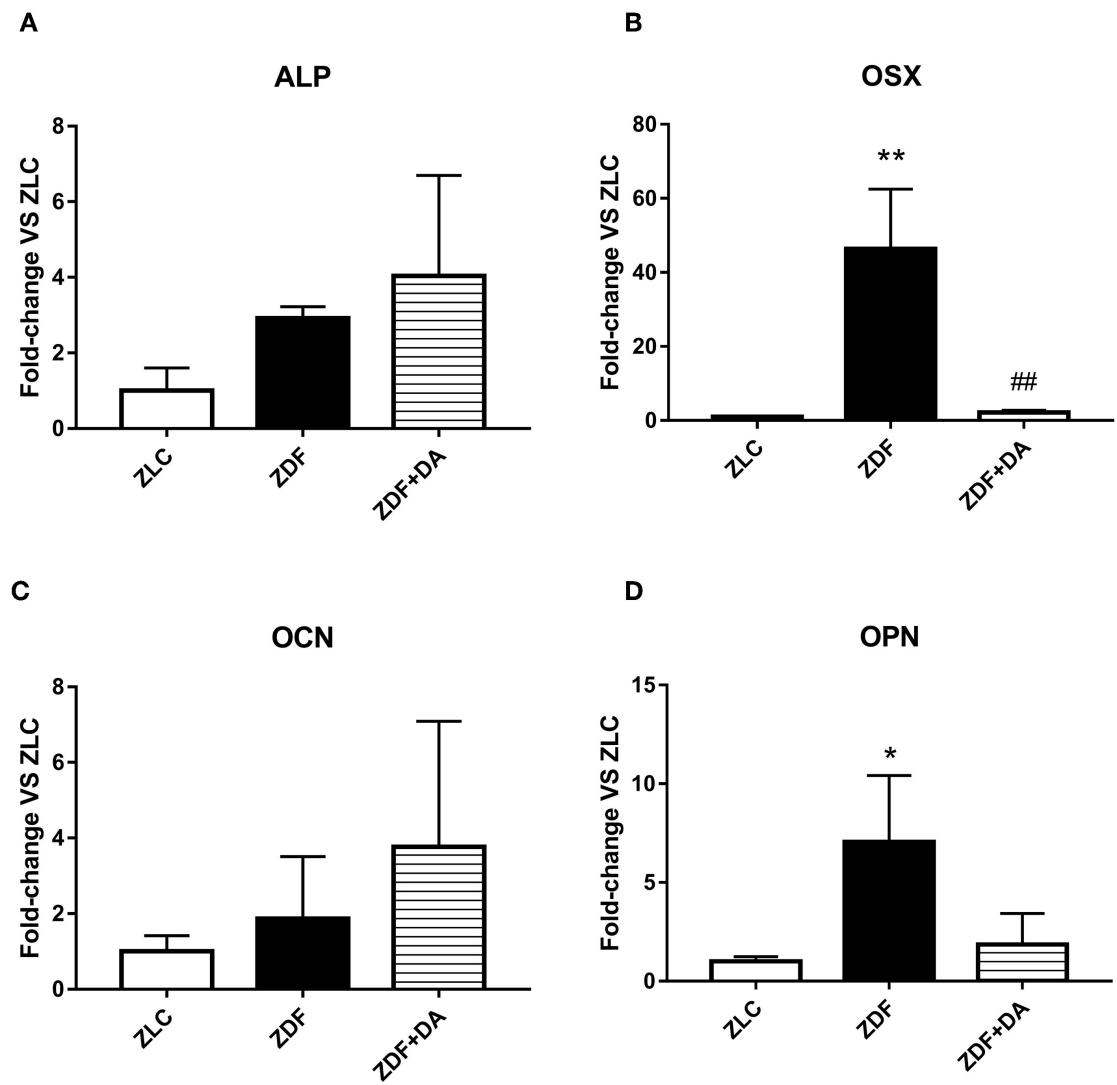
The expression of bone related genes. Twelve-week-old rats were randomly assigned into two subgroups: ZDF rats treated with vehicle (ZDF); ZDF rats treated with Dapagliflozin (ZDF+DA, Dapagliflozin, 1 mg/kg, po. per day). **(A)** ALP at the end of 9-week treatment. **(B)** OSX at the end of 9-week treatment. **(C)** OCN at the end of 9-week treatment. **(D)** OPN at the end of 9-week treatment. Values are means ± SD. Statistically significant differences compared to ZLC group are indicated with asterisk (*p < 0.05; **p < 0.01). Statistically significant differences compared to ZDF group are indicated with octothorpe (^##^*p* < 0.01). Abbreviations: ALP, alkaline phosphatase; OSX, Osterix; OCN, osteocalcin; OPN, osteopontin.

In order to exam whether diabetes or Dapagliflozin affects the expression of bone related genes, we selected four osteocytes related genes to put through a q-PCR test: alkaline phosphatase (ALP), markers that indicate the mineralization ability of osteoblasts (OBs); Osterix (OSX), a specific transcription factor of OBs which indicates the generation rate of OBs; osteocalcin (OCN), a marker of bone turnover; and osteopontin (OPN), an indication of osteocytes proliferation. As a result, the ZDF group demonstrated a significant increase in OSX and OPN compared to the ZLC group (Figure 8B, ZDF vs. ZLC, *p* < 0.01; Figure 8D, ZDF vs. ZLC, *p* < 0.05). After the treatment of Dapagliflozin, the generation of OBs was suppressed (Figure 8B, ZDF+DA vs. ZDF, *p* < 0.01). ALP and OCN demonstrated no significant change among the three groups (Figures 8A,C, *p* > 0.05).

## Discussion

Diabetes related osteoporosis, which contributes to an increased fracture risk, is a significant comorbidity in patients with either T1DM or T2DM, which is likely the consequence of many common variables, including chronic hyperglycemia (10). According to the work of Moayeri, an association between T2DM and overall fractures was found in patients, and these findings emphasize the need for fracture prevention strategies in patients with diabetes (11). In T1DM patients, hyperglycemia and impaired renal function were considered to be the main reasons of defections in bone structures. However, the situation in T2DM is more complicated. In our research, we choose ZDF rats over STZ add on high fat diets induced diabetic models, to study the effect of diabetes and SGLT2I on bone metabolism, because the pathologic characteristics of ZDF rats are more similar to T2DM (9).

One of our initial objectives of the project was to identify how diabetes works on bone metabolism. The results, as shown in Figures 3, 4, 6, indicate that diabetes can damage the mineral density of bone *via* hypercalciuria caused by increased levels of serum CT. Diabetes can damage bone in several ways (12–14), but in our study, interestingly, the loss of skeleton calcium in diabetic ZDF rats was mainly caused by hypercalciuria. ZDF rats demonstrated an increased level on serum CT, which is believed to be the reason of hypercalciuria and hyperphosphaturia. The same trend of CT was found in type 2 diabetes patients in Blasiak's study as well as in ours (15). A possible explanation for this might be that T2DM patients demonstrate the symptom of hyperinsulinemia. The beta cells in T2DM individuals tend to secrete more insulin in order to fight against hyperglycemia, this increased insulin secretion may cause intensive calcium uptake in beta cells thus lowering the level of serum calcium (16). Bone is the most important “calcium bank” in our bodies. When serum calcium drops, the activation of OCs will be boosted, causing enhanced bone resorption, releasing calcium into the blood to compensate for the serum calcium that dissipated during insulin secretion. As shown in Figures 3A,E, even though ZDF rats had higher UCCR, the serum calcium level demonstrated no significant difference compared to the matched controls. The process of bone resorption will increase serum phosphate simultaneously (15, 17). Serum phosphate levels are controlled primarily by the rate of proximal renal phosphate reabsorption (18). In order to prevent hyperphosphatemia, the secretion of CT is stimulated to inhibit the phosphate reabsorption (19). CT is a hormone that increases both calcium and phosphate exudation *via* the kidney and causes further wastage of the calcium stored in bone (20).

As a new of anti-diabetic drug class, the potential profits and risks of SGLT2I are not yet fully known. A number of clinical trials were carried out using different SGLT2I drugs and the results were heterogeneous. There are currently three SGLT2I drugs approved only for the treatment of T2DM: Canagliflozin, Dapagliflozin, and Empagliflozin. According to the clinical trials carried out by Kohan, canagliflozin and dapagliflozin showed different abilities in affecting Skeletal co-morbidities in T2DM patients (6), which indicated that the effect of SGLT2I on bone differed according to the specific choice of SGLT2I drug. The CANVAS study found out that after Canagliflozin treatment, fracture incidence increased significantly at limb (upper and lower) and insignificant changes were observed at other sites (e.g., the spine and thoracic cage) (21). A retrospective pooled dataset analysis from placebo-controlled, Phase III studies treating adult T2DM patients with either Canagliflozin or Dapagliflozin have identified modest, but clinically insignificant increases in serum phosphate, magnesium, osteocalcin, PTH and CTX-1 concentrations in patients treated with these SGLT2Is (22). In contrast, a pooled clinical data analysis for empagliflozin (11) examining *N* = 8500 T2DM patients from 17 placebo-controlled Phase I to III trials plus six extension trials (study duration up to 104 weeks), found no increase in fracture incidence and no significant change in serum calcium, phosphate, magnesium, parathyroid hormone, alkaline phosphatase, or urinary N-telopeptide concentration among empagliflozin-treated subjects, perhaps suggesting drug-specific differences in ultimate skeletal impact (23).

In our study, Dapagliflozin protects diabetic bones by resisting hyperglycemia and hyperinsulinemia. After treated with Dapagliflozin, blood glucose was lowered thus the calcium uptake caused by increased insulin secretion was prevented so that bone did not need to release calcium to fight against hypocalcemia. So far, there was no study designed to analyze the skeletal effect of SGLT2I on T2DM animal models. Two studies carried out by University of Kentucky Barnstable Brown Diabetes Center on STZ-induced DBA/2J mice demonstrated that Canagliflozin does not prevent diabetic bone disease (10, 23). However, the mice these two studies chose were induced into T1DM by directly reducing the amount of beta cells with STZ (24–26). Even though T1DM and T2DM share the similar characteristic of hyperglycemia, T2DM is a condition which is often complicated by insulin resistance, obesity, and secondary comorbidities which also indirectly influence skeletal homeostasis (23). This kind of mouse model dose not replicate the T2DM confounding variables such as hyperinsulinemia, nor does it replicate the impact of CT induced hypercalciuria on skeletal or clinical outcomes.

There were a number of studies aimed to explore the mechanism of how SGLT2I affects bone metabolism. Theoretically, SGLT2I can enhance urinary glucose excretion by inhibiting the function of the renal SGLT2 in the early proximal convoluted tubule. Glycosuria is likely to increased phosphate reabsorbed by the Na^+^-Phosphate transporter, thus elevating serum phosphate level, causing secondary hyperparathyroidism (17). Untreated hyperparathyroidism can cause hypercalcemia, hindering the process of calcification in osteoblasts (27). In this way, SGLT2I is suspected to have adverse skeletal effects by altering calcium and phosphate homeostasis which might lead to decline in bone density and an increased risk of bone fractures (7). However, in our study, PTH showed no change in ZDF+DA group compared to ZLC and ZDF groups (Figure 7F). Possible reasons were that the dosage and duration we chose were not enough to trigger such significant differences in PTH and serum phosphate or serum PTH level was suppressed by CT induced hyperphosphaturia in ZDF rats (23).

Besides the ability to resist hyperglycemia, we tend to study more direct effects of SGLT2I on osteocytes. However, although high affinity facilitative glucose transporters (GLUTs) are expressed in osteoblasts (GLUT-1 and GLUT-3) and osteoclasts (GLUT-1) (28, 29), SGLT2 has not been identified in osteocytes including OBs, OCs and MSCs (30), suggesting that a direct effect of Dapagliflozin on bone *via* SGLT2 is unlikely. Moreover, several studies showed that expression of SGLT2 is limited to the brush border membrane of the proximal tubule cells of the kidney in rodents (31) and humans (32, 33). These findings are consistent with the hypothesis that SGLT2I-related skeletal effects are more likely the result of systemic changes in bone-mineral homeostasis and hyperglycemia, rather than direct disruption of SGLT2 in osteocytes (23).

Our study demonstrated that Dapagliflozin, as an SGLT2I, has protective effects on bones of ZDF rats. However, a few limitations of this study must be recognized. First of all, the effects of Dapagliflozin on bones was only tested in ZDF rats, a T2DM model. Therefore, the effects of Dapagliflozin in animals with different metabolic profiles, such as OB/OB mouse or high fat diet add on STZ treatment models, may not be consistent with ZDF rats. Secondly, since we only tested one SGLT2I class drug at regular treatment dosage (based on 10 mg/day Dapagliflozin treatment for T2DM patients, the dosage for ZDF rats was converted *via* the Meeh-Rubner formula), it is unclear what dosage will benefit bone metabolism the most in the individuals with T2DM. As a new class of anti-diabetic drug, SGLT2I has drawn attention with its multiple therapeutic potential but the risk remained unclear due to the lack of animal and clinical data. Even though we conducted this study with ZDF rats to test skeletal effects of Dapagliflozin, there are still a number of differences between rodent and human in the physiology and metabolic profiles under the T2DM condition (T2DM patients often suffered from hyperuricacidemia while rodent do not). Therefore, the thus far results and controversies about SGLT2I treatment must be treated with cautious. Before the conclusion could be drawn, more clinical trials and animal researches are needed in order to figure out the difference in efficacy and safety between SGLT2Is. In conclusion, long-term normalization of hyperglycemia with Dapagliflozin is efficacious in preventing the occurrence of osteoporosis in T2DM. Moreover, as the application of SGLT2I has become more extensive, it is possible that the effects and mechanisms of Dapagliflozin on skeletal metabolism will be clarified by upcoming studies and data.

## Conclusion

In male ZDF rats, Dapagliflozin at doses of 1.0 mg/kg/day can suppress hyperglycemia, thus changing the serum CT level then alleviating the calcium loss *via* the kidney, hence preventing diabetic bone resorption. Our findings suggest that Dapagliflozin possesses protective effects toward bone in T2DM individuals and more studies should be carried out in order to clarify this theory before the expansion of clinical indications for SGLT2I therapy. For now, personalized assessment is crucial to patients with T2DM before the administration of this class of drug.

## Data Availability Statement

All datasets generated for this study are included in the manuscript/supplementary files.

## Ethics Statement

This study was carried out in accordance with the recommendations of Ethical code of laboratory animal, Southern Medical University Laboratory Animal Center. The protocol was approved by the ethics committee of Southern Medical University.

## Author Contributions

J-YW, Y-ZC, HC, HZ, and LY contributed to the design of the experiment. J-YW and S-LY researched data, contributed to the discussion, wrote the manuscript, reviewed, and edited the manuscript. J-YW provided statistical analyses, reviewed, and edited the manuscript. MA also reviewed and edited the manuscript.

### Conflict of Interest

The authors declare that the research was conducted in the absence of any commercial or financial relationships that could be construed as a potential conflict of interest.
