# Corrigendum: Dapagliflozin Attenuates Hyperglycemia Related Osteoporosis in ZDF Rats by Alleviating Hypercalciuria

**DOI:** 10.3389/fendo.2020.00524

**Published:** 2020-09-02

**Authors:** Ji-Yu Wang, Yan-Zhen Cheng, Shuang-Li Yang, Min An, Hua Zhang, Hong Chen, Li Yang

**Affiliations:** ^1^Department of Endocrinology and Metabolism, Zhujiang Hospital, Southern Medical University, Guangzhou, China; ^2^Department of Endocrinology and Metabolism, The Second Affiliated Hospital of GuiZhou Medical University, Kaili, China

**Keywords:** dapagliflozin, bone microarchitecture, ZDF rats, type 2 diabetes mellitus, Sodium dependent glucose transporters 2 inhibitor

In the original article, there was an error. The dosage of Dapagliflozin in *Abstract* should be 1.0 mg/kg/day. The period between digits “1” and “0” was accidentally removed.

A correction has been made to ***Abstract, Paragraph 1***:

“Recent studies showed that in patients with type 2 diabetes mellitus (T2DM), Sodium-dependent glucose transporters 2 inhibitor (SGLT2I) may cause potential adverse effects on skeleton such as increasing the risk of fracture. This risk is possibly mediated by effects induced by all SGLT2I class drugs but whether Dapagliflozin aggravates osteoporosis in patients with T2DM remains controversial. Therefore, we designed this study to explore how Dapagliflozin affects the metabolism and the quality of bone in. T2DM animal models The effect of Dapagliflozin on skeleton was evaluated on male ZDF (Zucker Diabetic Fatty) rats—a rat model of diet induced spontaneous T2DM. Dapagliflozin was administrated *via* gavage at the dosage of 1.0 mg/kg/day”.

**In the Discussion part, paragraph 7, we failed to describe how the dosage of dapagliflozin was determined**.

A correction has been made to ***Discussion, Paragraph 7***:

“Our study demonstrated that Dapagliflozin, as an SGLT2I, has protective effects on bones of ZDF rats. However, a few limitations of this study must be recognized. First of all, the effects of Dapagliflozin on bones was only tested in ZDF rats, a T2DM model. Therefore, the effects of Dapagliflozin in animals with different metabolic profiles, such as OB/OB mouse or high fat diet add on STZ treatment models, may not be consistent with ZDF rats. Secondly, since we only tested one SGLT2I class drug at regular treatment dosage (based on 10 mg/day Dapagliflozin treatment for T2DM patients, the dosage for ZDF rats was converted *via* the Meeh-Rubner formula), it is unclear what dosage will benefit bone metabolism the most in the individuals with T2DM. As a new class of anti-diabetic drug, SGLT2I has drawn attention with its multiple therapeutic potential but the risk remained unclear due to the lack of animal and clinical data. Even though we conducted this study with ZDF rats to test skeletal effects of Dapagliflozin, there are still a number of differences between rodent and human in the physiology and metabolic profiles under the T2DM condition (T2DM patients often suffered from hyperuricacidemia while rodent do not). Therefore, the thus far results and controversies about SGLT2I treatment must be treated with cautious. Before the conclusion could be drawn, more clinical trials and animal researches are needed in order to figure out the difference in efficacy and safety between SGLT2Is. In conclusion, long-term normalization of hyperglycemia with Dapagliflozin is efficacious in preventing the occurrence of osteoporosis in T2DM. Moreover, as the application of SGLT2I has become more extensive, it is possible that the effects and mechanisms of Dapagliflozin on skeletal metabolism will be clarified by upcoming studies and data”.

**In the original article, in the *Discussion* section, some citations for ref. 23 were missing**. The citations have now been inserted in *Discussion*, ***Paragraphs 3-6*** and should read:

“As a new of anti-diabetic drug class, the potential profits and risks of SGLT2I are not yet fully known. A number of clinical trials were carried out using different SGLT2I drugs and the results were heterogeneous. There are currently three SGLT2I drugs approved only for the treatment of T2DM: Canagliflozin, Dapagliflozin, and Empagliflozin. According to the clinical trials carried out by Kohan, canagliflozin and dapagliflozin showed different abilities in affecting Skeletal co-morbidities in T2DM patients [6], which indicated that the effect of SGLT2I on bone differed according to the specific choice of SGLT2I drug. The CANVAS study found out that after Canagliflozin treatment, fracture incidence increased significantly at limb (upper and lower) and insignificant changes were observed at other sites (e.g., the spine and thoracic cage) [21]. A retrospective pooled dataset analysis from placebo-controlled, Phase III studies treating adult T2DM patients with either Canagliflozin or Dapagliflozin have identified modest, but clinically insignificant increases in serum phosphate, magnesium, osteocalcin, PTH and CTX-1 concentrations in patients treated with these SGLT2Is [22]. In contrast, a pooled clinical data analysis for empagliflozin [11] examining *N* = 8500 T2DM patients from 17 placebo-controlled Phase I to III trials plus six extension trials (study duration up to 104 weeks), found no increase in fracture incidence and no significant change in serum calcium, phosphate, magnesium, parathyroid hormone, alkaline phosphatase, or urinary N-telopeptide concentration among empagliflozin-treated subjects, perhaps suggesting drug-specific differences in ultimate skeletal impact [23].

In our study, Dapagliflozin protects diabetic bones by resisting hyperglycemia and hyperinsulinemia. After treated with Dapagliflozin, blood glucose was lowered thus the calcium uptake caused by increased insulin secretion was prevented so that bone did not need to release calcium to fight against hypocalcemia. So far, there was no study designed to analyze the skeletal effect of SGLT2I on T2DM animal models. Two studies carried out by University of Kentucky Barnstable Brown Diabetes Center on STZ-induced DBA/2J mice demonstrated that Canagliflozin does not prevent diabetic bone disease [10, 23]. However, the mice these two studies chose were induced into T1DM by directly reducing the amount of beta cells with STZ [24–26]. Even though T1DM and T2DM share the similar characteristic of hyperglycemia, T2DM is a condition which is often complicated by insulin resistance, obesity, and secondary comorbidities which also indirectly influence skeletal homeostasis [23]. This kind of mouse model dose not replicate the T2DM confounding variables such as hyperinsulinemia, nor does it replicate the impact of CT induced hypercalciuria on skeletal or clinical outcomes.

There were a number of studies aimed to explore the mechanism of how SGLT2I affects bone metabolism. Theoretically, SGLT2I can enhance urinary glucose excretion by inhibiting the function of the renal SGLT2 in the early proximal convoluted tubule. Glycosuria is likely to increased phosphate reabsorbed by the Na^+^-Phosphate transporter, thus elevating serum phosphate level, causing secondary hyperparathyroidism [17]. Untreated hyperparathyroidism can cause hypercalcemia, hindering the process of calcification in osteoblasts [27]. In this way, SGLT2I is suspected to have adverse skeletal effects by altering calcium and phosphate homeostasis which might lead to decline in bone density and an increased risk of bone fractures [7]. However, in our study, PTH showed no change in ZDF+DA group compared to ZLC and ZDF groups (Figure 7F). Possible reasons were that the dosage and duration we chose were not enough to trigger such significant differences in PTH and serum phosphate or serum PTH level was suppressed by CT induced hyperphosphaturia in ZDF rats [23].

Besides the ability to resist hyperglycemia, we tend to study more direct effects of SGLT2I on osteocytes. However, although high affinity facilitative glucose transporters (GLUTs) are expressed in osteoblasts (GLUT-1 and GLUT-3) and osteoclasts (GLUT-1) [28, 29], SGLT2 has not been identified in osteocytes including OBs, OCs and MSCs [30], suggesting that a direct effect of Dapagliflozin on bone *via* SGLT2 is unlikely. Moreover, several studies showed that expression of SGLT2 is limited to the brush border membrane of the proximal tubule cells of the kidney in rodents [31] and humans [32, 33]. These findings are consistent with the hypothesis that SGLT2I-related skeletal effects are more likely the result of systemic changes in bone-mineral homeostasis and hyperglycemia, rather than direct disruption of SGLT2 in osteocytes [23].”

The authors apologize for these errors and state that this does not change the scientific conclusions of the article in any way. The original article has been updated.

